# The Effects of Non-Fiber Carbohydrate Content and Forage Type on Rumen Microbiome of Dairy Cows

**DOI:** 10.3390/ani11123519

**Published:** 2021-12-10

**Authors:** Zihai Wei, Xiao Xie, Mingyuan Xue, Teresa G. Valencak, Jianxin Liu, Huizeng Sun

**Affiliations:** 1 MoE Key Laboratory of Molecular Animal Nutrition, Institute of Dairy Science, College of Animal Sciences, Zhejiang University, Hangzhou 310058, China; weizihai365@163.com (Z.W.); myxue@zju.edu.cn (M.X.); teresavalencak@zju.edu.cn (T.G.V.); liujx@zju.edu.cn (J.L.); 2 School of Marine Sciences, Ningbo University, Ningbo 315800, China; Xiexiao@nbu.edu.cn

**Keywords:** non-fiber carbohydrate, alfalfa, low-quality forage, rumen microbiota, milk production

## Abstract

**Simple Summary:**

For sustainable development in the dairy industry, crop by-products play an important role, especially in areas that lack pasture and are not suitable for intensive cereal-adapted production (i.e., diets containing high amounts of cereal grains). However, feeding crop by-products usually lowers milk production in dairy cows due to their poor nutrient quality. In a previous study, we have demonstrated that non-fiber carbohydrate content (NFC) is a major limiting factor for the utilization of diets based on corn stover (CS). Although the higher abundance of easily fermentable energy in NFC and forage type can influence the synthesis of VFAs and MCP in the rumen and higher NFC content or high quality forages normally have a positive influence on the lactation performance of dairy cows, the underlying microbial mechanisms and potential effects on milk production are under-investigated to date. Here, we examined microbial composition and predicted the metabolism from cows fed CS-based diets with either high levels of NFC (H-NFC), or low levels of NFC (L-NFC). Control cows were fed an alfalfa-based diet (AH). Our results show that, supplementation of the CS-based diet with additional NFC increased amino acid biosynthesis in rumen microbiota in dairy cattle, and thus resulted in better nitrogen conversion. However, lower levels of fibrolytic capacity may limit dry matter intake of cows fed H-NFC and may prevent increased milk yield.

**Abstract:**

The main objective of our current study was evaluating the effects of NFC supplementation and forage type on rumen microbiota and metabolism, by comparing microbial structures and composition among samples collected from cows fed AH (alfalfa-based diet), H-NFC (CS-based diet with high NFC) and L-NFC (CS-based diet with low NFC) diets. Our results show that microbial communities were structurally different but functionally similar among groups. When compared with L-HFC, NFC increased the population of Treponema, Ruminobacter, Selenomonas and Succinimonas that were negatively correlated with ruminal NH3-N, and urea nitrogen in blood, milk and urine, as well as significantly increasing the number of genes involved in amino acid biosynthesis. However, when compared to the AH group, H-NFC showed a higher abundance of bacteria relating to starch degradation and lactate production, but a lower abundance of bacteria utilizing pectin and other soluble fibers. This may lead to a slower proliferation of lignocellulose bacteria, such as Ruminococcus, Marvinbryantia and Syntrophococcus. Lower fibrolytic capacity in the rumen may reduce rumen rotation rate and may limit dry matter intake and milk yield in cows fed H-NFC. The enzyme activity assays further confirmed that cellulase and xylanase activity in AH were significantly higher than H-NFC. In addition, the lower cobalt content in Gramineae plants compared to legumes, might have led to the significantly down-regulated microbial genes involved in vitamin B12 biosynthesis in H-NFC compared to AH. A lower dietary supply with vitamin B12 may restrict the synthesis of milk lactose, one of the key factors influencing milk yield. In conclusion, supplementation of a CS-based diet with additional NFC was beneficial for nitrogen conversion by increasing the activity of amino acid biosynthesis in rumen microbiota in dairy cattle. However, lower levels of fibrolytic capacity may limit dry matter intake of cows fed H-NFC and may prevent increased milk yield.

## 1. Introduction

Despite the fact that a majority of the world’s milk production is facilitated by grass- or cereal-based feeding regimes, crop-residue based feeding is still common in [1,2]. Crop residue is normally made up of the remainders of an agricultural crop after the harvest. Its advantages are availability and a low price, therefore being widely used as the main forage in many developing countries that lack pasture areas and cannot afford intensive cereal-based animal feed production. Corn stover (CS), as the most abundant crop by-product in China, has reached an annual production of 220 million tons, with a price of less than $100 per ton [3]. However, CS has higher lignocellulose but is lower in metabolic energy and crude protein (CP), when comparing its nutritional value with alfalfa hay (high-quality and commonly used in commercial farming) [4,5]. Using CS to replace alfalfa in dairy cow diets, even with similar dietary CP levels, significantly reduced the milk yield and milk protein contents [4,5]. Therefore, it is necessary to further investigate and develop strategies for improving CS utilization, thus alleviating the shortage of high-quality forage and promoting the development of dairy industry in disadvantaged, resource-poor countries.

We have found that the lower milk yield and milk protein contents in CS-fed cows compared to alfalfa-fed cows were partially due to the lower non-fiber carbohydrate (NFC) content in the CS containing diet [5]. NFCs provide sufficient energy for efficient microbial protein (MCP) synthesis [6]. Maximizing MCP synthesis increases the efficiency of N utilization and reduces N urinary excretion [7]. In our previous study, we evaluated lactation performance of dairy cows on a CS-based diet with an NFC concentration adjusted to the same level as the alfalfa-based diet [8]. The results showed that dairy cows fed a CS-based diet with NFC supplementation had significantly better feed efficiency (milk yield/dry matter intake) than those fed a CS-based diet with lower NFC content and had a similar feed efficiency to those fed an alfalfa-based diet (AH). NFC supplementation was beneficial for nitrogen conversion, indicating the importance of NFC supplementation for improved CS utilization. Nevertheless, even if the CS-based diet is supplemented with equal amounts of NFC with AH, significant differences in rumen metabolism, lowered dry matter intake and total milk yield were observed [8].

Rumen microbiota are directly involved in the degradation and metabolization of plant materials in the rumen, whilst providing the host with adequate metabolic energy and protein. Any change in nutrient availability will result in perturbed metabolic pathways distributed across a multitude of microbial populations [9]. The modification of nutrient sources affects microbiota and modifies the rumen ecosystem and host performance. Although the higher abundance of easily fermentable energy in NFC can influence carbohydrate and protein levels available in the rumen, and increase the synthesis of VFAs and MCP, the underlying microbial mechanisms and potential effects on milk production are under-investigated to date.

Thus, we aimed to explore the effects of NFC supplementation on rumen microbiota and metabolism, by comparing microbial structures and composition among samples collected from cows fed AH, H-NFC (CS-based diet with high NFC) and L-NFC (CS-based diet with low NFC) diets. We hypothesized that the differences in dietary NFC content may alter rumen microbial structure and composition affecting nitrogen and carbohydrate metabolism in dairy cows fed a CS containing diet, and may thus alter rumen fermentation, other physiological parameters, and ultimately milk production. Our work aims to provide novel approaches to optimizing the dietary utilization of crop-residue based diets in dairy cows.

## 2. Materials and Methods

### 2.1. Experimental Design

The samples used in this study were obtained in a previous animal experiment that has been described in Wei et al. [8]. A total of twelve Holstein cows in mid-lactation (159 ± 15 days in milk (DIM); 704 ± 72 kg of body weight; mean ± SD) and housed in a tie-stall barn were selected and assigned to a replicated 3 × 3 Latin square design with three diets: (1) low-NFC CS based diet (NFC = 35.6%, L-NFC), (2) high-NFCCS based diet (NFC = 40.1%, H-NFC), and (3) alfalfa hay-based diet (NFC = 38.9%, AH); the H-NFC was formulated to have a matched NFC content with the AH diet and both were higher than that of the L-NFC group. Each dietary treatment lasted for 21 days with the first 14 days as the adaptation phase. Swapping from one diet to another took place over four days, with a 25% daily increase of the new diet. The diets were offered three times a day at 07:00, 13:00 and 19:00 *ad libitum* while water was provided ad libitum for all animals. The cows were milked three times daily shortly after feeding at 08:00, 14:30 and 20:30, respectively. In both L-NFC and H-NFC, corn stover consisted of approximately 15% of total dry matter (DM) to replace alfalfa hay in AH for comparison. All the diets were composed to be isonitrogenous and isocaloric and to meet all requirements for milk production of 29 kg/day with 3.9% milk fat and 3.3% milk protein according to NRC [10]. All ingredients and the chemical composition of the three diets are listed in Supplementary Table S1.

### 2.2. Sample Collection and Measurements

The collection and chemical analyses of total mixed ration (TMR) samples, milk, blood and urine samples, dry matter intake (DMI) measurement and lactational performance including milk yield and milk composition were performed in our previous study where the details are given [8]. Rumen fluid samples were collected 3 h after the morning feeding on d 19 in each period according to Shen et al. [11]. Rumen fluid pH measurement, volatile fatty acids (VFA) concentration and ammonia N (NH3-N) concentration analysis were performed in our previous study [8], and triplicate 1-mL rumen fluid samples were frozen at −20 °C for later analyses of enzyme activity and rumen microbial DNA extraction. Rumen fluid activity of xylanase and CMCase was determined according to the dinitrosalicylic acid method [12]. All enzyme activities are expressed as μmol of decomposed monosaccharides released per minute and per milliliter of each sample.

### 2.3. Rumen Microbial DNA Extraction and 16S rRNA Gene Sequencing

DNA was isolated from rumen fluid samples using a QIAamp DNA Stool Mini Kit (QIAGEN, Düsseldorf, Germany). The quality and integrity of extracted DNA were assessed by nanodrop (ThermoFisher, Waltham, MA, USA) and gel electrophoresis. The hypervariable V3-V4 region was amplified using the bacterial universal primer set 341F/806R [13] (341F: 5′-ACTCCTACGGGRSGCAGCAG-3′, 806R: 5′-GGACTACVVGGGTATCTAATC-3′) in the Phusion High-Fidelity PCR MaterMix (New England Biolabs, Ipswich, MA, USA). The PCR products were verified using 2% gel electrophoresis and were purified using the QIAquick Gel Extraction Kit (QIAGEN, Düsseldorf, Germany). After library construction, all samples were sequenced on the Illumina HiSeq platform for pair-end reads of 250 bp.

### 2.4. Bioinformatic Processing and Taxonomic Annotation

Adaptors were trimmed from 3′ end of demultiplexed raw reads using Cutadapt [14] in pair-end mode. Pair-end reads without Ns and maximum number of expected errors smaller than 2 were retained. Reads at the first instance of a quality scoring less than or equal to 2 were truncated as default suggestion in the dada2 pipeline [15]. Error rates of each sample were estimated by self-consistent non-supervised machine-learning for true sequence variance inference proposed by dada2. Merging of sequences was performed by aligning the denoised pair-end reads with overlapping by at least 15 bases. The amplicon sequence variable table was constructed, and chimeric sequences were identified and discarded from the table. The unique amplicon representative sequences were then processed in QIIME2 and classified taxonomically using the q2-feature-classifer against a SILVA release 138 database for small subunit ribosomal RNAs [16]. ASVs (amplicon sequence variants) belonging to chloroplasts and mitochondria were filtered as contaminants, and archaea were separated from the data. A phylogenetic tree was constructed based on the alignment of represented sequences using MUSCLE and FasTree [17,18].

### 2.5. Analysis of Microbial Variation and Functional Prediction, and Statistical Analysis

The ASV abundance of tabular, corresponding taxonomic data, phylogenetic tree and grouping information, were imported to create a phyloseq object in R [19] using the phyloseq package for later analysis [20]. The alpha-diversity indices, including Shannon, Simpson, Chao1 and observed species from each sample, were calculated, and the differences among dietary groups were analyzed using linear models with diet and subject as the main factors. Multiple comparisons were tested by the Waller–Duncan k-ratio test adjusted with a false discovery rate by using the package agricolae [21]. Beta-diversity was calculated by either Bray–Curtis dissimilarity or weighted unifrac distance, and microbial communities with higher similarity were clustered in non-metric multidimensional scaling (NMDS). All figures were constructed using the package *ggplot2*. The differences among the three diets were tested using the adonis2 algorithm in the package “vegan” [22]. Pairwise comparisons were made using permutation ANOVAs adjusted by false discovery rate on the distance matrix.

The contribution of each single species to the respective bacterial communities in each group was assessed based on the effect size calculated by linear discriminant analysis of effect size algorithm (LEfSe) [23]. More comparisons of other abundant bacteria in the dietary groups were performed by the analysis of composition of microbiota (ANCOM) [24] on all phylogenetic levels. The resulting relative abundance matrix of significantly different abundant bacteria were later normalized by z-scores and variation was visualized on a heatmap plot using the package “pheatmap” [25]. Bacteria within each phylogenetic level were clustered according to the unweighted pair-group method with arithmetic means (UPGMA). In addition, intersections of differently abundant genera are listed in a Venn diagram.

The correlations between nutrient components, rumen fermentation parameters and physiological indexes including DMI, milk yield (MY), feed efficiency (FE), milk fat content (MF), milk total solids content (TS), concentration of milk urea nitrogen (MUN), blood urea nitrogen (BUN) and urine nitrogen (UN), and urine volume (UV) with bacterial community distance matrices were tested using the package ade4 [26]. Redundancy analysis (RDA) was used to investigate the relationship between microbial community and change in physiological indexes and rumen fermentation parameters. It was performed after the introduction of a community matrix of significantly changed genera, and physiological indexes and rumen fermentation parameters were entered as environmentally constrained variables. After fitting the environmental vectors onto the ordination, the importance of each factor on the changing microbial community was obtained [22]. In addition, Spearman correlations were computed, and the resulting correlation matrixes were visualized on heatmaps.

To better understand the dietary induced, functional changes in the microbiota, a functional prediction was performed using PICRUSt2 based on 16S rRNA gene sequence data [27]. The generated ASVs were used to predict gene family abundances and pathway abundances were calculated. The PCA plot and Kruskal–Wallis test for pairs of treatment groups were then performed by STAMP [28]. Statistical significance of all analyses was declared at a *p* value ≤ 0.05 with highly significant values at *p* ≤ 0.01, and trends were declared at 0.05 < *p* value ≤ 0.10.

## 3. Results

### 3.1. Structure of Dominant Rumen Bacterial Communities

The 16S rRNA gene sequencing generated a total of 2,758,492 sequences, with 78,814 ± 1008 reads per sample. After quality filtering, sample inference, merging of paired reads and removing chimeras, raw sequences were clustered into 10,940 distinct bacterial variants according to the dada2 algorithm. By annotating against the SILVA database, a total of 17 bacterial phyla were identified at the phylum level, with Bacteroidetes (60.2%), Firmicutes (34.3%), Spirochaetes (1.85%) and Proteobacteria (1.68%) being the most prevalent, followed by Patescibacteria (0.73%), Actinobacteria (0.58%) and Cyanobacteria (0.33%) (Supplementary Figure S1). Sequences annotated as archaea, chloroplasts or mitochondria were discarded from the downstream analysis. At genus level, genera with relative abundances > 0.10% and prevalences higher than 50% of the animals were considered predominant core microbiota, including *Prevotella*, Rikenellaceae_RC9 gut group, Oscillospiraceae NK4A214 group, Christensenellaceae R−7 group, *Ruminococcus*, *Acetitomaculum*, *Treponema*, Prevotellaceae UCG-001, Lachnospiraceae NK3A20 group and *Saccharofermentans* (Supplementary Figure S2).

### 3.2. Rumen Bacterial Diversity Compared between Cows Fed Three Different Diets

**Figure 1:**
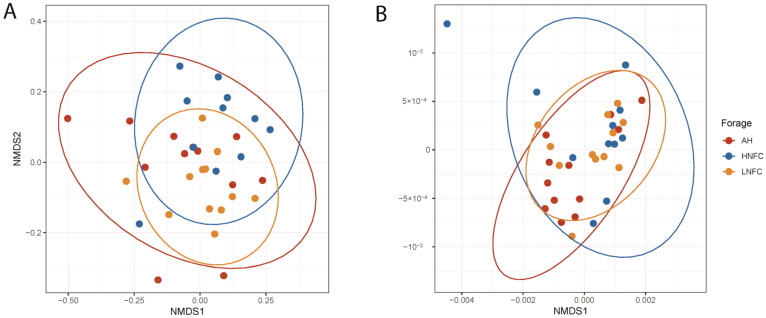
NMDS of beta-diversity calculated based on (**A**) Bray–Curtis dissimilarity and (**B**) weighted unifrac distance. Samples were obtained from different dietary treatments and are indicated with differently shaped symbols and colors. The colored ellipses are described by a 95% confidence interval.

**Figure 2:**
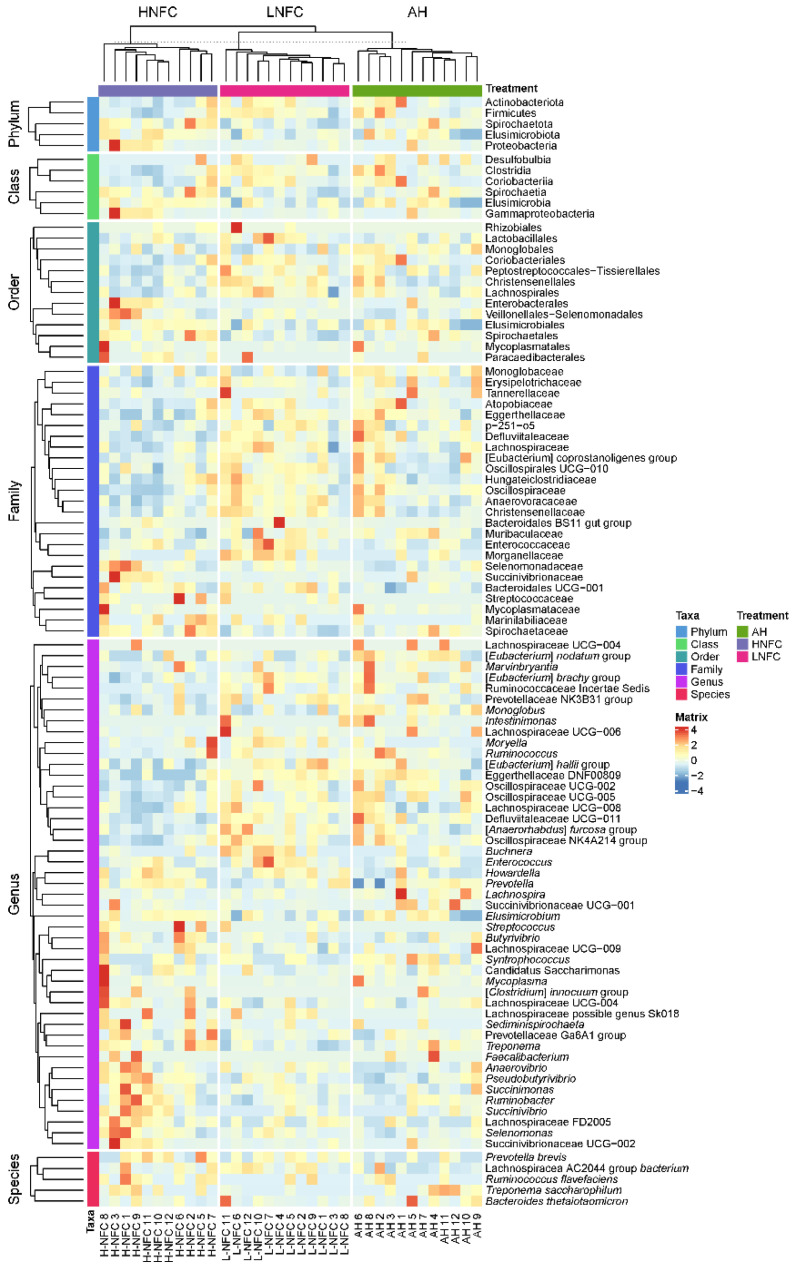
Rumen microbial community variation in three dietary treatments. The columns of the heatmap represent the samples collected from each animal, and rows correspond to the bacteria that significantly changed under the influence of dietary treatments at different phylogenetic levels. The relative abundance was normalized by z-scores across different samples. Taxa are separately clustered at different phylogenetic levels and dietary groups.

Alpha diversity of the bacterial communities from each dietary group was not significantly different (*p* > 0.05) between the observed species, the ASV-level richness index (Chao1) and diversity indices (Shannon and Simpson) (Table 1). According to non-metric multidimensional scaling analysis, which is given in Figure 1, the microbial communities varied across different animals without clear separation among the three dietary treatments. The unweighted pair group method with an arithmetic mean (UPGMA) dendogram based on Bray–Curtis dissimilarity distance showed that the L-NFC and AH groups clustered more together than the H-NFC group (Figure 2). The result was further confirmed by pairwise permutation multivariate analysis of variance (MANOVA) showing that the diets had a significant impact on the structure of bacterial communities (*p* = 0.01) and that the bacterial community in the H-NFC group was significantly different from that observed both in the L-NFC group and the AH group (both *p* = 0.03). No difference was observed between the L-NFC and the AH group. However, on the basis of weighted unifrac distance incorporating phylogenetic relations between species, the difference among the groups was not significant (*p* > 0.05). As for intra-group individual differences, calculating ‘betadipers’ in the package “vegan” showed that the bacterial communities were more dispersed across different individuals within the AH group, followed by H-NFC and L-NFC (Supplementary Figure S3). This result is confirmed by the NMDS plots showing that the confidence interval ellipse of AH was larger than in the other two groups (Figure 1).

### 3.3. Differential Rumen Bacterial Taxa and Enzyme Activities from Cows Fed Different Diets

**Figure 3:**
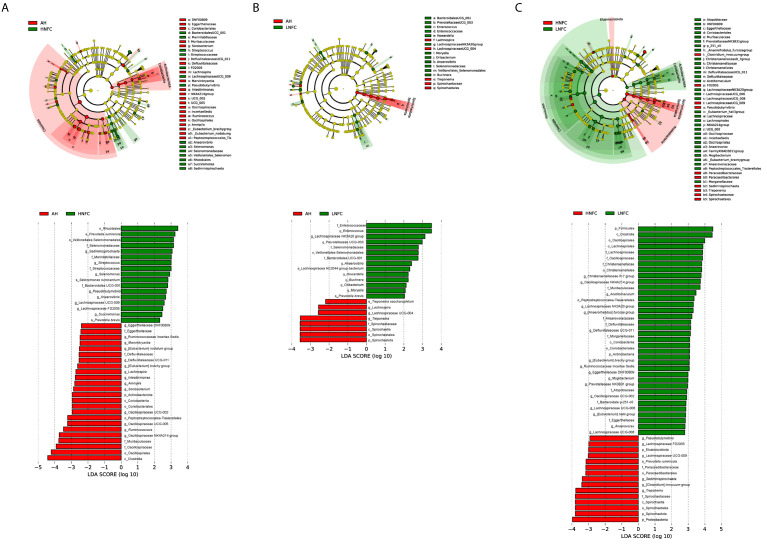
Linear discriminant analysis (LDA) between microbiota from (**A**) alfalfa based diet (AH) and a CS based diet with high NFC (H-NFC); (**B**) AH and CS based diet with low NFC (L-NFC); (**C**) H-NFC and L-NFC. Differences are represented by color of the group, where taxa are most abundant. Histogram of LDA scores computed for each taxon ranging from phylum to species. The LDA scores represented the difference in relative abundance with exponent fold change of 10 between two communities.

**Figure 4:**
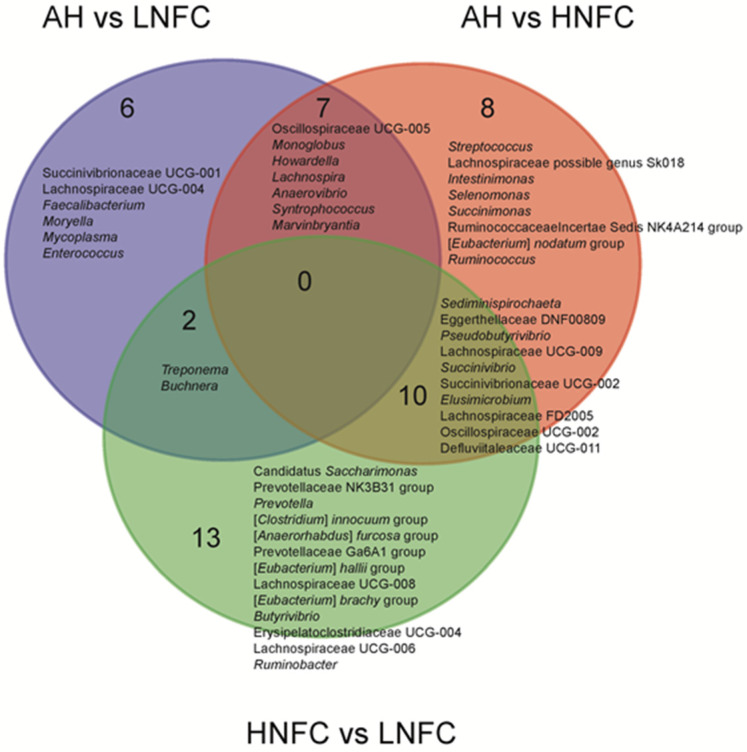
Significantly different abundant genera among dietary treatments using ANCOM. The names and the number of interactions and unique genera are listed in each area of the Venn diagram.

The LEfSe analyses, comparing two dietary groups at one time, H-NFC and L-NFC (Figure 3A), H-NFC and AH groups (Figure 3B) and L-NFC and AH groups (Figure 3C), identified 5 and 23, 1 and 22, 1 and 11 differential phyla and genera, respectively. AH, H-NFC and L-NFC were enriched with bacteria belonging to Coriobacteriia, Spirochaetia, and Clostridia, respectively. These bacteria detected in LEfSe analysis contributed most to the observed differences among the three groups. Additional differential comparisons with ANCOM analyses showed that the relative abundance of 47 genera were significantly different between dietary groups. The variation in microorganisms across diets at the phylum level, class, order, family and genus are presented in Figure 2. Of the genera that significantly differed between dietary treatments, seven genera (Oscillospiraceae UCG-005, *Monoglobus*, *Howardella*, *Lachnospira*, *Anaerovibrio*, *Syntrophococcus* and *Marvinbryantia*) were significantly different in the AH group compared to both the L-NFC and the H-NFC group, as a result of changing main forage resources (Figure 4). Two genera, *Treponema* and *Buchnera* were significantly different in the L-NFC group compared to both the AH and the H-NFC group, possibly due to the low NFC content. Ten genera (*Sediminispirochaeta*, Eggerthellaceae DNF00809, *Pseudobutyrivibrio*, Lachnospiraceae UCG-009, *Succinivibrio*, Succinivibrionaceae UCG-002, *Elusimicrobium*, Lachnospiraceae FD2005, Oscillopsiraceae UCG-002, Defluviitaleaeceae UCG-001) were significantly different in the H-NFC compared to both the AH and L-NFC group. The changes in rumen bacteria with the activity levels in lignin, cellulose, hemicellulose, pectin and starch utilization are presented in Supplementary Figure S4. Both CMCase and xylanase catalytic activities were measured and are presented in Table 2. The activities were significantly higher in the AH and L-NFC groups compared to the H-NFC (*p* < 0.05).

### 3.4. Correlation of Nutrient Ingredients, Physiological Indexes and Rumen Fermentation with Bacterial Communities

**Figure 5:**
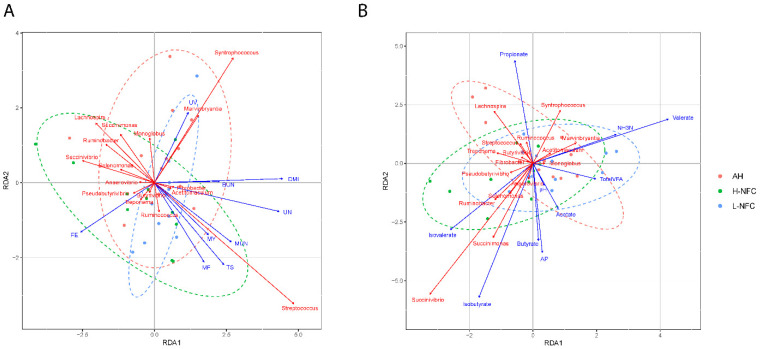
Canonical correlation analysis of microbial community relating to (**A**) physiological indexes and (**B**) rumen fermentation variables. The rumen fermentation variables and physiological indexes are indicated by solid blue lines, and the 15 differentially abundant genera are indicated by solid red lines. Samples collected from different dietary treatment groups are presented in different colors.

Judging from the Mantel statistic calculated from Spearman’s rank correlation, rumen fermentation significantly correlated with bacterial communities (*p* < 0.01). However, the correlation between physiological indexes and microbiota was not significant (*p* = 0.93). RDA was used to further explore the correlations between each parameter and rumen microbiota at genus level. The RDA results showed that the microbial community was significantly influenced by DMI (*p* < 0.01), crude ash (CA) content (*p* = 0.03), Acidic detergent fiber (ADF, *p* = 0.02), neutral detergent fiber (NDF, *p* = 0.05) and NFC (*p* = 0.03), and was correlated with the concentration of urine nitrogen (UN, *p* < 0.01), ruminal fermentation of propionate (*p* < 0.01), butyrate (*p* = 0.04), isovalerate (*p* < 0.01), valerate (*p* < 0.01), isovalerate (*p* = 0.02) and the acetate/propionate ratio (*p* = 0.01) (Figure 5, Supplementary Table S2). Together, these results indicate that DMI and the composition of dietary carbohydrate were the predominant influencing factors for the bacterial community, and lead to changes in urine nitrogen and ruminal fermentation.

**Figure 6:**
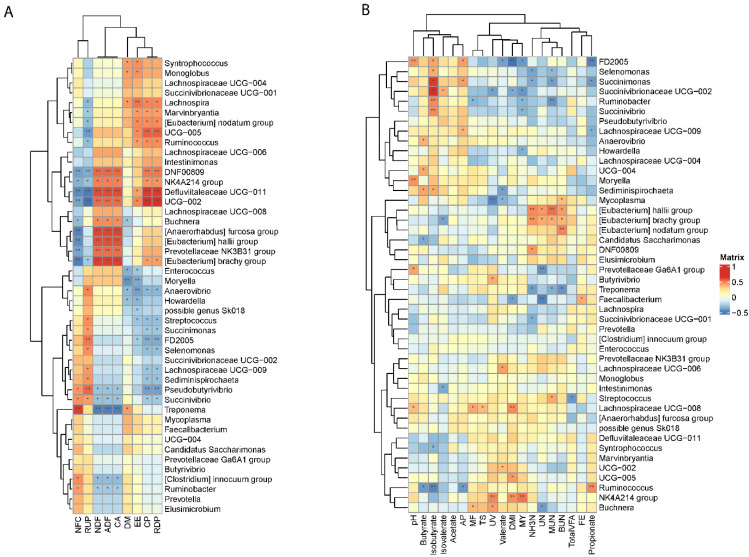
Spearman correlations of differentially abundant genera and (**A**) nutrient components, (**B**) physiological indexes and rumen fermentation variables. Columns represent nutrient components, physiological parameters and rumen fermentation variables, and rows correspond to the genera significantly affected by the dietary treatments. Gradient colors indicate correlation coefficients. Correlation with *p* ≤ 0.01 were marked with **, and 0.01 < *p* ≤ 0.05 were marked with *. Genera were clustered according to Euclidean distances calculated based on the correlation coefficients.

The RDA results together with the Spearman correlation analysis revealed the correlation of each significantly changed genus with nutrient composition, physiological indexes and rumen fermentation parameters (Figure 6). The correlation analysis identified that NFC was positively correlated with *Treponema*, *Ruminobacter*, [*Clostridium*] *innocuum*, and *Pseudobutyrivibrio*, *Succinivibrio*; however, these genera were negatively correlated with the content of NDF, ADF and CA in the diet (*p* < 0.05). Although genera including *Ruminococcus*, *Lachnospira*, *Marvinbryantia*, and the [*Eubacterium*] *nodatum* group were not correlated with NFC, they were significantly affected by the content of rumen-degradable protein (RDP) and CP in the diet (*p* < 0.05). Defluviitaleaceae UCG−011, Oscillospiraceae UCG−002, Eggerthellaceae DNF00809, Prevotellaceae NK4A214 group, *Buchnera*, [*Anaerorhabdus*] *furcosa* group, [*Eubacterium*] *hallii* group, Prevotellaceae NK3B31 group and [Eubacterium] *brachy* group were all positively correlated with NDF and ADF levels in the diet, and most genera were significantly positively correlated with RDP and CP levels in the diet (*p* < 0.05).

As for the rumen fermentation and physiological indexes, nitrogen metabolism (NH_3_-N, UN, MUN, BUN) was negatively correlated with *Treponema*, *Succinimonas*, *Selenomona*, *Ruminobacter*, Prevotellaceae Ga6A1 group and *Faecalibacterium*, but was positively correlated with [*Eubacterium*] *hallii* group, [*Eubacterium*] *brachy* group, [*Eubacterium*] *nodatum* group, *Mycoplasma*, Eggerthellaceae DNF00809, *Streptococcus* (*p* < 0.05). For the bacteria that significantly correlated with nitrogen metabolism, *Treponema*, *Succinimonas*, *Selenomona*, *Ruminobacter* and Prevotellaceae Ga6A1 group were significantly higher in H-NFC compared to L-NFC. DMI, which is one of the predominant factors influencing the structure of the bacterial community, was positively correlated with Lachnospiraceae UCG−008, Oscillospiraceae UCG−005 and Prevotellaceae NK4A214 group, but was negatively correlated with *Faecalibacterium*, Succinivibrionaceae UCG−002 and FLachnospiraceae FD2005 (*p* < 0.05).

### 3.5. Functional Prediction and Microbial Metabolism under the Effect of Dietary Treatment

**Figure 7:**
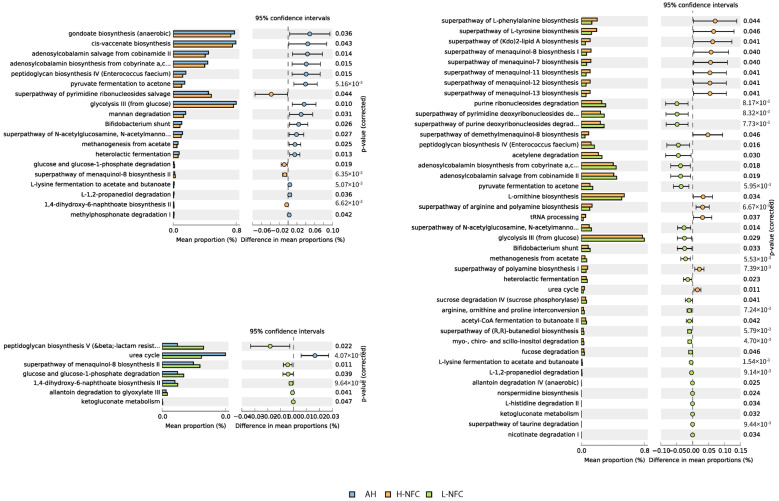
Predicted metagenomic difference between alfalfa based diet (AH) and a CS based diet with high NFC (H-NFC); AH and CS based diet with low NFC (L-NFC); H-NFC and L-NFC. Pathways were sorted in descending order based on the affecting factor.

To elucidate the functional profile of rumen microbiota under the influence of dietary treatments, PICRUSt2 was used to analyze and predict the functional capabilities of bacteria. The ASVs were finally assigned to 1960 enzyme classification numbers that belong to 389 MetaCyc pathways. PCA analysis demonstrated that there was no clear separation of the three dietary groups, indicating similar bacterial functionality (Supplementary Figure S5). Relating to nitrogen metabolism, H-NFC was significantly enriched with genes involved in amino acid synthesis (superpathway of L-phenylalanine biosynthesis, superpathway of L-tyrosine biosynthesis, L-ornithine biosynthesis and superpathway of arginine and polyamine biosynthesis) compared to L-NFC (Figure 7, *p* < 0.05). In addition, the number of genes relating to the urea nitrogen cycle in L-NFC was significantly lower than that in both the AH and H-NFC groups, but the number of genes relating to allantoin degradation was significantly higher (*p* < 0.05). Comparing between AH and H-NFC, one of the major differences attributed to the size effect was that H-NFC was lower in genes involved in the metabolism of adenosylcobalamin (adenosylcobalamin salvage from cobinamide II, adenosylcobalamin biosynthesis from cobyrinate a,c-diamide I), which is the precursor of Vitamin B12.

## 4. Discussion

### 4.1. Effect of Plant Sources and Nutritional Composition on Rumen Microbial Diversity

Diet is one of the most important factors regulating rumen microbiota [9]. In our study, *Acetitomaculum*, Christensenelleae R-7 group, Lachnospiraceae NK3A20 group, Oscilospiraceae NK4A214 group, Prevotellaceae UCG-001, Rikenellaceae RC9 gut group, *Ruminicoccus*, Succinivibrionaceae UCG-002 and *Treponema* were identified as ’core’ taxa in ruminal fermentation across all dietary treatments, indicating their essential role in occupying niches in rumen ecology and diet degradation on feeds that are made of similar plant sources.

Supplementing additional NFC resulted in identical main nutrient content in H-NFC and AH, while being composed of CS as the main forage. Theoretically, under the influence of both forage type and nutrient composition, microbial community structures in H-NFC should be similar to L-NFC and AH. However, according to the analysis of PCoA and adonis2, calculated based on Bray–Curtis distance, microbial communities of H-NFC were significantly different from the other two, while AH and L-NFC more closely resembled each other. When the similarity distance was replaced by the weighted unifrac metric, which incorporates phylogenetic distances and the quality of each feature between observed organisms in the computation, the differences between microbial communities were no longer significant. The comparison between the groups on predicated metagenomic information with PCA analysis and permutation MANOVAs further confirmed the absence of significant differences at functional levels. Despite overall similarity among dietary treatment groups, a total of 47 genera were significantly different between dietary groups according to the ANCOM analysis. In comparison to bacteria that are important for carbohydrate metabolism, AH was highly abundant with *Lachnospira* and *Monoglobus*, two bacteria utilizing pectin as their main carbon source [29,30]. In contrast, bacteria relating to starch degradation and lactate production were significantly increased in the H-NFC compared to the AH group, with *Prevotella ruminicola*, *Succinimonas*, *Streptococcus and Selenomonas ruminantium* being the most prevalent species. Our results suggest an inherent difference in carbohydrate metabolism caused by the influence of forage nutrients. This can be explained by the microbial redundancy in rumen ecology, where each substrate or linkage within biopolymers can become metabolized or attacked by multiple, coexisting, taxonomically distinct organisms [31]. As was shown in our study, functional difference was not significant among treatment groups according to PICRUSt functional prediction data. The redundancy might therefore lead to different microbial composition but with the closed ecosystem functionality in our study. Ecological modification by various plant sources changed the overall microbial structure, while the similar nutrition maintained the functional group shifted from one functional equivalent status to another.

### 4.2. NFC Composition as Important Factor Affecting Rumen Microbial Composition and Carbohydrate Metabolism

The carbohydrate structure of cytoplasm and forage cell walls are biologically complex, consisting of cellulose, hemicellulose, pectins, galactans, β-glucans and phenolic lignins in the cell wall, as well as starch, sugars, organic acids and fructan in the cytoplasmic fraction [32]. Pectins that are located in the cell wall are considered structural carbohydrates but are soluble in neutral detergent solution and are readily digested by rumen microbes [33]. The cell walls of dicotyledonous plants, especially the leguminous forage, contain large amounts of non-cellulose polysaccharides, particularly pectin and xyloglucan, with pectin consisting of approximately 45% NFC in alfalfa hay [34]. In contrast, the monocotyledons of the Gramineae contain relatively low amounts of pectin and xyloglucan, but large quantities of heteroxylcans and (1, 3; 1, 4)-β-D-glucan [35]. It has been demonstrated that differences in dietary starch and pectin result in altered fermentation, digestion and milk production of the animal [36]. Studies have observed improved microbial synthesis [37] and an influx of microbial crude protein into the small intestine [38] as well as an improved animal performance [39] in response to supplemented pectin.

AH was abundant with higher numbers of *Ruminococcus* and *Marvinbryantia* than in the H-NFC group. *Ruminococcus* is highly efficient in cellulose and hemicellulose degradation. The latter one is member of Clostridia and ferments amorphous cellulose but not crystalline cellulose [40]. The enzyme activity assays further showed that the cellulase and xylanase activities in the AH group were significantly higher than in H-NFC (Table 2), indicating that there might be a higher fibrolytic capacity in AH than H-NFC. In addition, the AH group also showed signs of lignin degradation. *Syntrophococcus*, highly abundant in AH, is the prevalent species capable of O demethylation of methoxylated lignin monoaromatic derivatives in the rumen [41]. Breakdown of chemical linkages between carbohydrates and phenolic compounds of lignin are beneficial for the adhesion of microorganisms and accelerated cellulose degradation [42]. According to the study of the colonization process of a bacterial community on the forage surface, during forage incubation in the rumen, there was a transition from primary to secondary, in which *Prevotella* and *Succinivbrio,* which are highly abundant in the initial stage, were decreased and gradually replaced by *Fibrobacter*, *Treponema*, *Ruminicoccus* and *Butyrivibrio* that were specialized in cellulose and hemicellulose fermentation [43]. Since AH and H-NFC are equivalent in ADF content, a higher abundance of lignocellulose bacteria possibly suggests that microbiota in AH were shifting to faster lignocellulose metabolism than in H-NFC during bacterial colonization, and there might be a higher fibrolytic capacity in AH than H-NFC.

*Ruminococcus* are able to produce the pectin methylesterase, pectin lyase and polygalacturonases [44], and utilize pectin from alfalfa to stimulate its uptake in the rumen [45]. Its abundance was positively correlated with the pectin content in the diet. Similar results were obtained in the study from Zhao et al. [46], in which decreasing NDF and the copy number of *Ruminococcus* were greater for pectin than starch. Huhtanen [38] reported greater rumen and total track digestibility of NDF when cannulated cattle were fed beet pulp-containing diets as compared to barley-based (high starch) diets. A high content of pectin in alfalfa [34] might result in faster proliferation of lignocellulose bacteria in AH. A lower abundance of lignocellulose bacteria in H-NFC may also relate to detrimental effects caused by the over population of amylolytic bacteria and excessive starch degradation. Starch can be rapidly fermented to lactic acid and lowers the pH in the rumen, which is detrimental to the fibrolytic capacity [47]. Since H-NFC had a similar pH but was significantly lower in total VFAs compared to AH [8], cows fed H-NFC probably accumulated more lactic acid. The accumulation of lactic acid may reduce the rumen pH faster than VFAs. The enriched population of amylolytic bacteria and the lower abundance of lignocellulose bacteria at reduced fibrolytic capacity in H-NFC might decrease rumen NDF digestibility in H-NFC, and thus might restrict rumen rotation rate and might limit DMI and total milk yield in cows fed H-NFC.

### 4.3. NFC Supplementation Shifts Rumen Microbial Metabolism towards Amino Acids Biosynthesis

One of the major drawbacks of feeding cows with TMRs based on CS as the main nutrient is that these diets are typically high in rumen-degradable protein but low in non-structural carbohydrates, causing microbes to use protein as an energy source and resulting in excess rumen ammonia-nitrogen [48]. Excess ammonia enters the liver through the blood, participates in the ornithine cycle and then synthesizes urea. This was the case in the L-NFC group whose UN and BUN were significantly higher, and the oversupplying of RDP relative to rumen-undegradable protein increased the MUN concentrations greater than 18 mg/dL [8]. Higher concentrations of urea in the blood could increase the amount of urea flowing back into the rumen via saliva and the rumen wall. Consequently, we observed significantly increased levels of *Howardella* in L-NFC, a gram-positive bacterium that is strongly uricolytic and generates ATP through the hydrolysis of urea [49]. The improved nitrogen conversion efficiency in H-NFC was related to the increased abundance of bacteria, such as *Treponema*, *Ruminobacter*, *Selenomonas* and *Succinimonas*, which were negatively correlated with ruminal ammonia, and urea nitrogen in blood, milk and urine. *Treponema* can only use ammonia in the rumen as a nitrogen source, and higher abundances are beneficial to improve the nitrogen conversion efficiency in the rumen [50].

In addition, according to results from the predicted metagenome, there were significantly fewer genes in L-NFC involved in the urea cycle pathway but more genes were involved in allantoin degradation compared to both the AH and H-NFC groups. Since the urea cycle is restricted to ureotelic organisms, mapping of genes to the urea cycle is simply due to bacteria containing distant homologous enzymes with important roles in mammalian pathways [27]. Nevertheless, the distinction of the two metabolic pathways can shed light on the flux of ammonia nitrogen, in which amino groups donated by ammonia are incorporated into L-arginine and L-ornithine in the urea cycle [51], while ammonia is completely liberated through allantoin degradation by bacteria under anaerobic conditions [52]. Metagenomic data also confirmed that more genes are involved in the amino acid biosynthesis pathway in H-NFC compared to L-NFC, including pathways of L-phenylalanine, L-tyrosine, L-ornithine, arginine and polyamine biosynthesis. No significant difference was observed in amino acid metabolism between AH and H-NFC. These results were in line with the animal study, showing that NFC supplementation in a CS-based diet had a significant effect on promoting the synthesis of microbial amino acids, reducing N emission and thus improving the nitrogen conversion rate of diet [8].

### 4.4. AH-Based Diet Was Beneficial for Microbial Synthesis of Vitamin B12

Another major difference with regards to microbial functionality compared between AH and H-NFC is the biosynthesis of adenosylcobalamin. Vitamin B12 is essential as a cofactor of methylmalonyl-CoA isomerase, an enzyme necessary for the use of propionic acid that is produced in large quantities in highly productive dairy cows for the synthesis of milk lactose [53]. The requirements for vitamin B12 in dairy cows are relatively high (0.34–0.68 μk/kg BW). However, the efficiency of production of vitamin B12 by ruminal microorganisms and absorption efficiency in dairy cows are very low [54]. Therefore, supplementation of vitamin B12 or cobalamin precursors are beneficial for milk production in dairy cows [55,56]. Especially for ruminants feeding on poor-quality forages, supplementing cobalt can enhance the digestibility of low quality forage [53]. A higher abundance of genes relating to vitamin B12 biosynthesis in AH were attributed to the high content of dietary cobalt that is essential for nodulation and nitrogen fixation in legumes [57]. Legumes, such as alfalfa or clover, represent the main sources of cobalt in the natural diet of some ruminants [54]. In addition, diet composition and the forage: concentrate ratio play a fundamental role in the production efficiency of vitamin B12. The synthesis of vitamin B12 is positively associated with dietary concentrations of NDF and ADF, and is negatively correlated with the concentration of starch in the diet [58,59]. Except for the difference in cobalt, legume species are much richer in macroelements than grasses growing under comparable conditions. Trace elements, in particular I, Cu, Zn, Co and Ni, are also generally higher in legumes than in grasses grown in temperate climates [60], some of which are important for the productivity of dairy cows. Therefore, when using CS to replace alfalfa hay as the main forage for TMR, an adequate trace element supply has to be considered.

## 5. Conclusions

Compared with L-NFC, feeding a CS-based diet supplemented with NFC increased the relative abundance of *Treponema, Ruminobacter, Selenomonas and Succinimonas,* which are highly efficient in ruminal ammonia utilization. A functional prediction demonstrated that genes involved in amino acid biosynthesis in H-NFC were significantly increased. However, when compared to AH, microbiota in H-NFC were significantly lowered in fibrolytic bacteria, including *Ruminococcus*, *Marvinbryantia* and *Syntrophococcus*, and decreased the enzyme activity of CMCase and xylanase. In addition, the lower cobalt content in Gramineae plants compared to legumes, might result in significantly down-regulated microbial genes involved in vitamin B12 biosynthesis in H-NFC compared to AH. A lower supply with vitamin B12 may possibly restrict the synthesis of milk lactose, one of the key factors in milk yield. Based on the above results, we propose to increase the proportion of pectin in NFC and at the same time to supplement a cobalamin precursor for improving vitamin B12 microbial synthesis when feeding cows with a CS based diet to achieve a higher milk production. Our results provide novel insights into the understanding of rumen microbial mechanisms relating to NFC content and forage type. Our results also offer a potential strategy for enhancing the utilization of a CS based diet for improved milk production in dairy cows.

## Figures and Tables

**Table 1:** Alpha diversity of the bacterial community in the rumen of dairy cows exposed to three different diets.

Diversity Index	Treatment ^1^			SEM	*p* Value
	AH	L-NFC	H-NFC		
Observed species	1483	1485	1453	22.0	0.53
Chao1	1668	1619	1619	29.7	0.41
Shannon	8.30	8.31	8.25	0.07	0.79
Simpson	0.9900	0.9907	0.9903	0.0009	0.87

^1^ AH = TMR containing alfalfa hay; L-NFC = TMR containing CS with a low content of NFC; H-NFC = TMR containing CS formulated to match NFC concentration with the AH diet.

**Table 2:** Enzyme activity of rumen microorganisms exposed to three different diets.

Enzyme Activity (U/mL)	Treatment ^1^			SEM	*p* Value
	AH	L-NFC	H-NFC		
CMCase ^2^	0.383 ^a^	0.366 ^a^	0.294 ^b^	0.017	<0.01
Xylanase	1.557 ^a^	1.639 ^a^	1.352 ^b^	0.069	0.039

^1^ AH = TMR containing alfalfa hay; L-NFC = TMR containing CS with a low content of NFC; H-NFC = TMR containing CS formulated to match NFC concentration with the AH diet. ^2^ CMCase, Carboxymethyl cellulase. ^a,b^ Means with different superscript letter differ (*p* < 0.05) among the dietary treatments.

## Data Availability

The datasets generated for this study can be found in the NCBI sequence read archive (SRA), accession number PRJNA771787.

## Supplementary Materials

The following are available online at https://www.mdpi.com/article/10.3390/ani11123519/s1, Figure S1: Relative abundance of bacteria at (A) phylum and (B) genus level. The top 10 phyla and genera were repsented using different colors. Figure S2: The core microbiome of animal under the effect of different dietary traetments. Figure S3: Analysis of similarities presented intra-group distance and inter-group distance. Figure S4: Boxplot of relative abundace of 13 differential abundant genera. The abundace of bacteria were log10 transferred. Samples from different dietary treatments were represented by different colors. Figure S5: Principle component analysis of predicted metagnomic data. Samples from different dietary treatments were represented by different colors. Table S1: Ingredients and chemical composition of the 3 experimental diets. Table S2: Statistical result by fitting the environmental vectors onto microbiota ordination.

## References

[1] Knips V. (2005). Developing Countries and the Global Dairy Sector Part I: Global Overview. https://www.fao.org/3/bp204e/bp204e.pdf.

[2] Walli T.K., Garg M.G., Makkar H.P.S. (2012). Crop Residue Based Densified Total Mixed Ration—A User-Friendly Approach to Utilise Food Crop By-Products for Ruminant Production.

[3] Jiang Y., Zhang J., Nan Z., Wang L. (2016). Competitiveness analysis of alfalfa international trade in China. Pratacult. Sci..

[4] Zhu W., Fu Y., Wang B., Wang C., Ye J.A., Wu Y.N., Liu J.-X. (2013). Effects of dietary forage sources on rumen microbial protein synthesis and milk performance in early lactating dairy cows. J. Dairy Sci..

[5] Wang B., Mao S.Y., Yang H.J., Wu Y.M., Wang J.K., Li S.L., Shen Z.M., Liu J.X. (2014). Effects of alfalfa and cereal straw as a forage source on nutrient digestibility and lactation performance in lactating dairy cows. J. Dairy Sci..

[6] Shabi Z., Arieli A., Bruckental I., Aharoni Y., Zamwel S., Bor A., Tagari H. (1998). Effect of the Synchronization of the Degradation of Dietary Crude Protein and Organic Matter and Feeding Frequency on Ruminal Fermentation and Flow of Digesta in the Abomasum of Dairy Cows. J. Dairy Sci..

[7] Thomas P.C. (1973). Microbial protein synthesis. Proc. Nutr. Soc..

[8] Wei Z.H., Zhang B.X., Liu J.X. (2018). Effects of the dietary nonfiber carbohydrate content on lactation performance, rumen fermentation, and nitrogen utilization in mid-lactation dairy cows receiving corn stover. J. Anim. Sci. Biotechnol..

[9] Newbold C.J., Ramos-Morales E. (2020). Review: Ruminal microbiome and microbial metabolome: Effects of diet and ruminant host. Animal.

[10] NRC (2001). Nutrient Requirements of Dairy Cattle.

[11] Shen J.S., Chai Z., Song L.J., Liu J.X., Wu Y.M. (2012). Insertion depth of oral stomach tubes may affect the fermentation parameters of ruminal fluid collected in dairy cows. J. Dairy Sci..

[12] Bailey M.J., Biely P., Poutanen K. (1992). Interlaboratory testing of methods for assay of xylanase activity. J. Biotechnol..

[13] Wang Y., Qian P.Y. (2009). Conservative Fragments in Bacterial 16S rRNA Genes and Primer Design for 16S Ribosomal DNA Amplicons in Metagenomic Studies. PLoS ONE.

[14] Martin M. (2011). Cutadapt removes adapter sequences from high-throughput sequencing reads. EMBnet J..

[15] Callahan B.J., Mcmurdie P.J., Rosen M.J., Han A.W., Johnson A.J., Holmes S.P. (2016). DADA2: High-resolution sample inference from Illumina amplicon data. Nat. Methods..

[16] Quast C., Pruesse E., Yilmaz P., Gerken J., Schweer T., Yarza P., Peplies J., Glöckner F.O. (2013). The SILVA ribosomal RNA gene database project: Improved data processing and web-based tools. Nucleic Acids Res..

[17] Price M.N., Dehal P.S., Arkin A.P. (2009). FastTree: Computing Large Minimum Evolution Trees with Profiles instead of a Distance Matrix. Mol. Biol. Evol..

[18] Toth F., Frank N., Elliott S.B., Geor R.J., Boston R.C. (2004). MUSCLE: Multiple sequence alignment with high accuracy and high throughput. Nucleic Acids Res..

[19] R Core Team (2021). R: A Language and Environment for Statistical Computing. https://www.r-project.org.

[20] McMurdie P.J., Holmes S. (2013). Phyloseq: An R Package for Reproducible Interactive Analysis and Graphics of Microbiome Census Data. PLoS ONE.

[21] De Mendiburu F. (2019). Agricolae: Statistical Procedures for Agricultural Research. https://cran.r-project.org/web/packages/agricolae/index.html.

[22] Dixon P. (2003). VEGAN, a package of R functions for community ecology. J. Veg. Sci..

[23] Segata N., Izard J., Waldron L., Gevers D., Miropolsky L., Garrett W.S., Huttenhower C. (2011). Metagenomic biomarker discovery and explanation. Genome Biol..

[24] Mandal S., Van Treuren W., White R.A., Eggesbø M.Å., Knight R.T., Peddada S.D. (2015). Analysis of composition of microbiomes: A novel method for studying microbial composition. Microb. Ecol. Health Dis..

[25] Kolde R. (2018). Pheatmap: Pretty Heatmaps. http://github.com/raivokolde/pheatmap.

[26] Dray S., Dufour A.-B. (2007). Theade4Package: Implementing the Duality Diagram for Ecologists. J. Stat. Softw..

[27] Douglas G.M., Maffei V.J., Zaneveld J.R., Yurgel S.N., Brown J.R., Taylor C.M., Huttenhower C., Langille M.G.I. (2020). PICRUSt2 for prediction of metagenome functions. Nat. Biotechnol..

[28] Parks D.H., Tyson G.W., Hugenholtz P., Beiko R.G. (2014). STAMP: Statistical analysis of taxonomic and functional profiles. Bioinformatics.

[29] Kim C.C., Kelly W.J., Patchett M.L., Tannock G.W., Jordens Z., Stoklosinski H.M., Taylor J.W., Sims I.M., Bell T.J., Rosendale D.I. (2017). Monoglobus pectinilyticus gen. nov., sp. nov., a pectinolytic bacterium isolated from human faeces. Int. J. Syst. Evol. Microbiol..

[30] Duskova D., Marounek M. (2001). Fermentation of pectin and glucose, and activity of pectin-degrading enzymes in the rumen bacterium Lachnospira multiparus. Lett. Appl. Microbiol..

[31] Weimer P.J. (2015). Redundancy, resilience, and host specificity of the ruminal microbiota: Implications for engineering improved ruminal fermentations. Front. Microbiol..

[32] Hall M.B., Hoover W.H., Jennings J.P., Webster T.K.M. (1999). A method for partitioning neutral detergent-soluble carbohydrates. J. Sci. Food Agr..

[33] Van Soest P.J. (1994). Nutritional Ecology of the Ruminant.

[34] Martin N., Mertens D. (2005). Reinventing alfalfa for dairy cattle and novel uses. Proceedings: California Alfalfa and Forage Symposium.

[35] Carpita N.C., Gibeaut D.M. (1993). Structural models of primary cell walls in flowering plants: Consistency of molecular structure with the physical properties of the walls during growth. Plant J..

[36] Leiva E., Hall M.B., Van Horn H.H. (2000). Performance of Dairy Cattle Fed Citrus Pulp or Corn Products as Sources of Neutral Detergent-Soluble Carbohydrates. J. Dairy Sci..

[37] Zhao X.H., Liu C.J., Liu Y., Li C.Y., Yao J.H. (2012). Effects of replacing dietary starch with neutral detergent-soluble fibre on ruminal fermentation, microbial synthesis and populations of ruminal cellulolytic bacteria using the rumen simulation technique (RUSITEC). J. Anim. Physiol. Anim. Nutr..

[38] Huhtanen P. (1988). The effects of barley, unmolassed sugar-beet pulp and molasses supplements on organic matter, nitrogen and fibre digestion in the rumen of cattle given a silage diet. Anim. Feed. Sci. Technol..

[39] Kim S.C., Adesogan A.T., Arthington J.D. (2007). Optimizing nitrogen utilization in growing steers fed forage diets supplemented with dried citrus pulp. J. Anim. Sci..

[40] Wolin M.J., Miller T.L., Collins M.D., Lawson P.A. (2003). Formate-Dependent Growth and Homoacetogenic Fermentation by a Bacterium from Human Feces: Description of *Bryantella formatexigens* gen. nov., sp. nov. Appl. Environ. Microbiol..

[41] Dore J., Bryant M.P. (1990). Metabolism of One-Carbon Compounds by the Ruminal Acetogen *Syntrophococcus sucromutans*. Appl. Environ. Microbiol..

[42] Akin D. (1988). Biological structure of lignocellulose and its degradation in the rumen. Anim. Feed. Sci. Technol..

[43] Huws S.A., Edwards J.E., Creevey C., Stevens P.R., Lin W., Girdwood S.E., Pachebat J., Kingston-Smith A.H. (2015). Temporal dynamics of the metabolically active rumen bacteria colonizing fresh perennial ryegrass. FEMS Microbiol. Ecol..

[44] Pettipher G.L., Latham M.J. (1979). Characteristics of Enzymes Produced by Ruminococcus flavefaciens which Degrade Plant Cell Walls. Microbiology.

[45] Gradel C.M., Dehority B. (1972). Fermentation of isolated pectin and pectin from intact forages by pure cultures of rumen bacteria. Appl. Microbiol..

[46] Zhao X.H., Gong J.M., Zhou S., Fu C.B., Liu C.J., Xu L.J., Pan K., Qu M.R. (2015). Effects of Degradable Protein and Non-Fibre Carbohydrates on Microbial Growth and Fermentation in the Rumen Simulating Fermenter (Rusitec). Ital. J. Anim. Sci..

[47] Calsamiglia S., Blanch M., Ferret A., Moya D. (2012). Is subacute ruminal acidosis a pH related problem? Causes and tools for its control. Anim. Feed. Sci. Technol..

[48] Bach A., Calsamiglia S., Stern M.D. (2005). Nitrogen Metabolism in the Rumen. J. Dairy Sci..

[49] Cook A.R., Riley P.W., Murdoch H., Evans P., McDonald I. (2007). *Howardella ureilytica* gen. nov., sp. nov., a Gram-positive, coccoid-shaped bacterium from a sheep rumen. Int. J. Syst. Evol. Microbiol..

[50] Liu J., Pu Y.-Y., Xie Q., Wang J.-K., Liu J.-X. (2015). Pectin Induces an In Vitro Rumen Microbial Population Shift Attributed to the Pectinolytic Treponema Group. Curr. Microbiol..

[51] Jackson M.J., Beaudet A.L., O’Brien W.E. (1986). Mammalian Urea Cycle Enzymes. Annu. Rev. Genet..

[52] Serventi F., Ramazzina I., Lamberto I., Puggioni V., Gatti R., Percudani R. (2010). Chemical Basis of Nitrogen Recovery through the Ureide Pathway: Formation and Hydrolysis of S-Ureidoglycine in Plants and Bacteria. ACS Chem. Biol..

[53] McDowell L.R. (2000). Vitamins in Animal and Human Nutrition.

[54] Brewer K., Maylin G., Fenger C., Tobin T. (2016). Cobalt use and regulation in horseracing: A review. Comp. Exerc. Physiol..

[55] Girard C., Matte J. (2005). Effects of Intramuscular Injections of Vitamin B12 on Lactation Performance of Dairy Cows Fed Dietary Supplements of Folic Acid and Rumen-Protected Methionine. J. Dairy Sci..

[56] Preynat A., Lapierre H., Thivierge M.C., Palin M.F., Matte J.J., DesRochers A., Girard C.L. (2009). Influence of methionine supply on the response of lactational performance of dairy cows to supplementary folic acid and vitamin B12. J. Dairy Sci..

[57] Bakken A.K., Synnes O.M., Hansen S. (2004). Nitrogen fixation by red clover as related to the supply of Cobalt and Molybdenum from some Norwegian soils. Acta Agric. Scand. Sect. B.

[58] Akins M.S., Bertics S.J., Socha M.T., Shaver R.D. (2013). Effects of cobalt supplementation and vitamin B12 injections on lactation performance and metabolism of Holstein dairy cows. J. Dairy Sci..

[59] Beaudet V., Gervais R., Graulet B., Nozière P., Doreau M., Fanchone A., Castagnino D.D.S., Girard C.L. (2016). Effects of dietary nitrogen levels and carbohydrate sources on apparent ruminal synthesis of some B vitamins in dairy cows. J. Dairy Sci..

[60] López-Alonso M. (2012). Trace Minerals and Livestock: Not Too Much Not Too Little. ISRN Vet. Sci..

