# Supplementary material for: The Effects of Non-Fiber Carbohydrate Content and Forage Type on Rumen Microbiome of Dairy Cows
Source: Animals (Basel). 2021 Dec 10;11(12):3519. doi: 10.3390/ani11123519 (PMC8698165; doi:10.3390/ani11123519)
Supplement: Supplementary file 1 [file animals-11-03519-s001.zip › Supplementary Figures.pdf]

Supplementary Figures

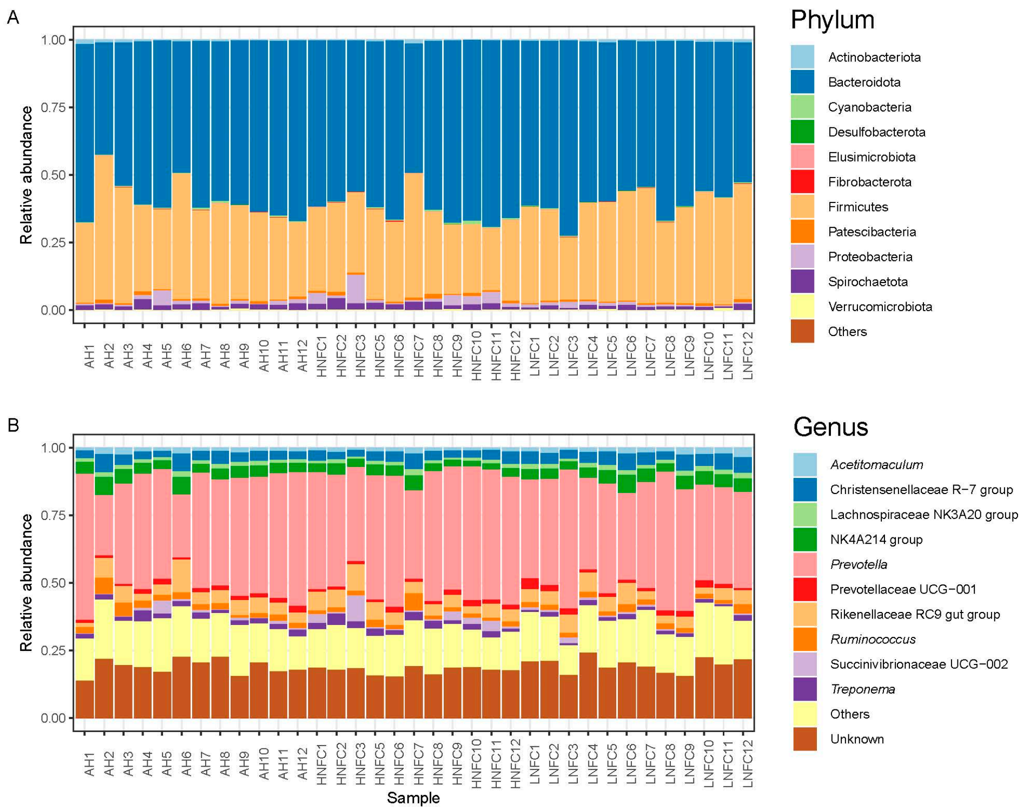

Supplementary Figure S1. Relative abundance of bacteria at (A) phylum and (B) genus level. The top 10 phyla and genera were repsented using different colors.

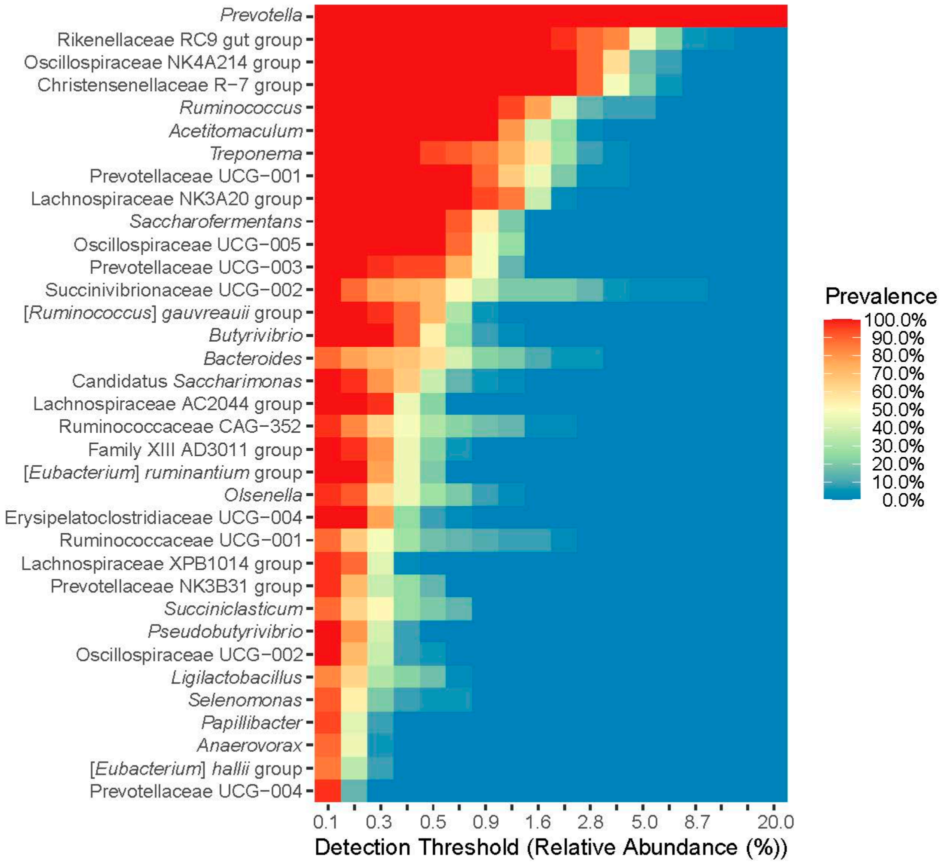

Supplementary Figure S2. The core microbiome of animal under the effect of different dietary traetments.

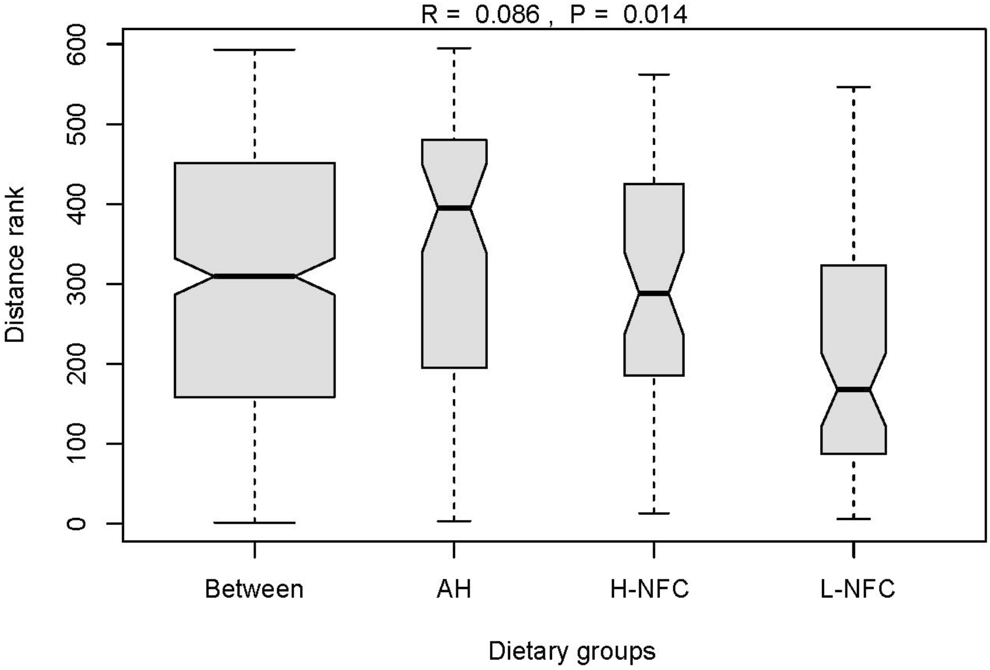

Supplementary Figure S3. Analysis of similarities presented intra-group distance and inter-group distance.

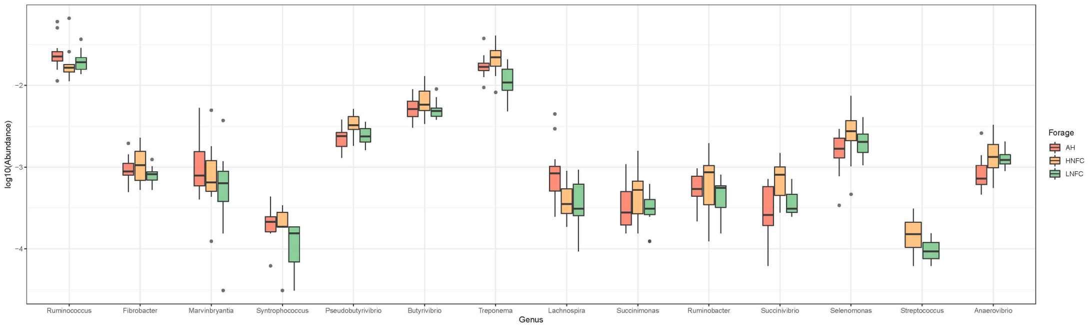

Supplementary Figure S4. Boxplot of relative abundace of 13 differential abundant genera. The abundace of bacteria were log10 transferred. Samples from different dietary treatments were represented by different colors.

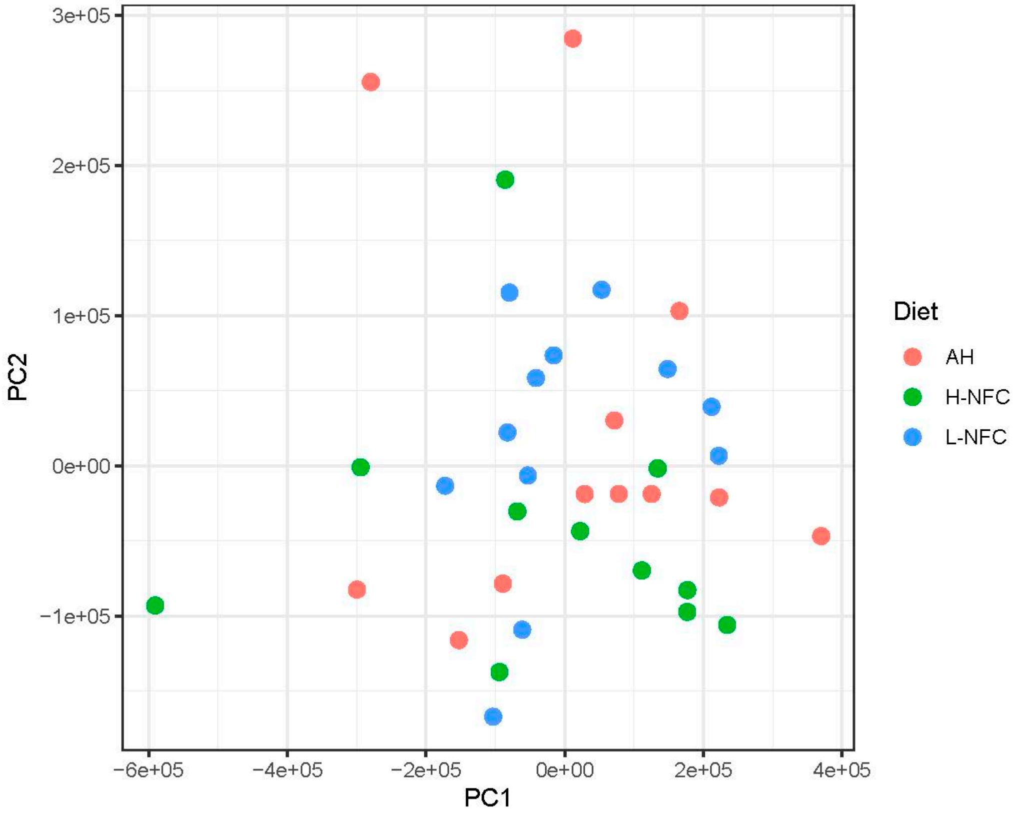

Supplementary Figure S5. Principle component analysis of predicted metagnomic data. Samples from different dietary treatments were represented by different colors.
