# Supplementary material for: The Effects of Non-Fiber Carbohydrate Content and Forage Type on Rumen Microbiome of Dairy Cows
Source: Animals (Basel). 2021 Dec 10;11(12):3519. doi: 10.3390/ani11123519 (PMC8698165; doi:10.3390/ani11123519)
Supplement: Supplementary file 1 [file animals-11-03519-s001.zip › Supplementary Tables.pdf]

Supplementary Tables

Supplementary Table S1. Ingredients and chemical composition of the 3 experimental diets

Items	Treatment[a]		
	AH	L-NFC	H-NFC
Dietary ingredient, g/kg of DM			
Alfalfa hay	149.0	0.0	0.0
Oat hay	49.5	44.1	46.8
Corn stover	0.0	147.0	148.0
Corn silage	267.0	257.0	266.0
Ground corn grain	297.0	229.0	299.0
Wheat bran	49.5	103.0	5.4
Soybean meal	99.0	177.0	175.0
Cottonseed meal	49.5	4.9	21.2
Premix[b]	39.6	38.2	39.4
Chemical composition[c], % of DM			
Dry matter	46.2	46.4	45.5
Crude protein	15.0	14.9	14.5
RDP, % of Crude protein	64.2	63.3	62.1
RUP, % of Crude protein	35.8	36.7	37.9
Ether extracts	3.8	3.6	3.6
Crude ash	7.7	8.3	7.5
Neutral detergent fiber	34.6	37.5	34.3
Acid detergent fiber	19.5	19.9	19.0
Non-fiber carbohydrate[c]	38.9	35.6	40.1
NE_L, MJ/kg	6.73	6.57	6.69
Diet price[d], $/kg DM	0.388	0.353	0.360

This table is cited from Wei et al. (2018)

[a]AH = Total mixed rations containning alfalfa hay; L-NFC = Total mixed rations containing corn stover with low content of non-fiber carbohydrates; H-NFC = Total mixed rations containing corn stover with high content of non-fiber carbohydrates similar to AH.

[b]Contained (per kilogram of DM): 250,000 IU of vitamin A, 50,000 IU of vitamin D, 1,100 IU of vitamin E, 600 mg of Fe, 650 mg of Cu, 3,000 mg of Zn, 630 mg of Mn, 17 mg of Se, 36 mg of I, 8 mg of Co, 150-180 g of salt, water ≤100 g

[c]Non-fiber carbohydrate = 100 – % neutral detergent fiber − % crude protein − % ether extract − % ash; RDP (rumen-degradable protein), RUP (rumen-undegradable protein) and NEL were estimated according to CPM dairy 3.0

[d]Diet price was calculated based on the ingredient purchase prices by the farm

Supplementary Table S2. Statistical result by fitting the environmental vectors onto microbiota ordination

Constrains	R2	P value[1]
**Diet composition**		
Dry matter	0.004	0.929
Crude protein	0.124	0.134
RDP	0.068	0.343
RUP	0.068	0.342
Ether extracts	0.000	0.997
Crude ash	0.206	0.027*
Acidic detergent fiber	0.223	0.022*
Neutral detergent fiber	0.182	0.045*
Non-fiber carbohydrate	0.208	0.028*
Starch	0.154	0.073
Sugar	0.056	0.411
**Physiological indexes**		
Dry matter intake	0.276	0.007**
Milk yield	0.073	0.274

Feed efficiency	0.114	0.157
Milk fat	0.096	0.199
Total solid	0.139	0.086
Milk urine nitrogen	0.130	0.115
Blood urine nitrogen	0.071	0.321
Urine nitrogen	0.268	0.006**
Urine volume	0.063	0.355
**Rumen fermentation parameters**		
pH	0.016	0.792
Ammonia nitrogen	0.120	0.121
Acetate	0.069	0.312
Propionate	0.326	0.006**
Butyrate	0.185	0.036*
Isobutyrate	0.607	0.001**
Valerate	0.307	0.002**
Isovalerate	0.235	0.023*
Total VFA	0.056	0.412
Acetate/Propionate ratio	0.243	0.012*

[1] $P \leq 0.01$ were marked with **, and $0.01 < P \leq 0.05$ were marked with *
